# A blood-brain barrier-penetrating AAV2 mutant created by a brain microvasculature endothelial cell-targeted AAV2 variant

**DOI:** 10.1016/j.omtm.2023.02.016

**Published:** 2023-03-02

**Authors:** Hayato Kawabata, Ayumu Konno, Yasunori Matsuzaki, Hirokazu Hirai

**Affiliations:** 1 Department of Neurophysiology & Neural Repair, Gunma University Graduate School of Medicine, Maebashi, Gunma 371-8511, Japan; 2 Viral Vector Core, Gunma University, Initiative for Advanced Research, Maebashi, Gunma 371-8511, Japan

**Keywords:** AAV, PHP.B, PHP.eB, BR1, blood-brain barrier, capsid, neutralizing antibody, transcytosis, C57BL/6 mouse, BALB/c mouse

## Abstract

Upon systemic administration, adeno-associated virus serotype 9 (AAV9) and the capsid variant PHP.eB show distinct tropism for the central nervous system (CNS), whereas AAV2 and the capsid variant BR1 transduce brain microvascular endothelial cells (BMVECs) with little transcytosis. Here, we show that a single amino acid substitution (from Q to N) in the BR1 capsid at position 587 (designated BR1N) confers a significantly higher blood-brain barrier (BBB) penetration capacity to BR1. Intravenously infused BR1N showed significantly higher CNS tropism than BR1 and AAV9. BR1 and BR1N likely use the same receptor for entry into BMVECs; however, the single amino acid substitution has profound consequences on tropism. This suggests that receptor binding alone does not determine the final outcome *in vivo* and that further improvements of capsids within predetermined receptor usage are feasible.

## Graphical abstract

**Figure:**
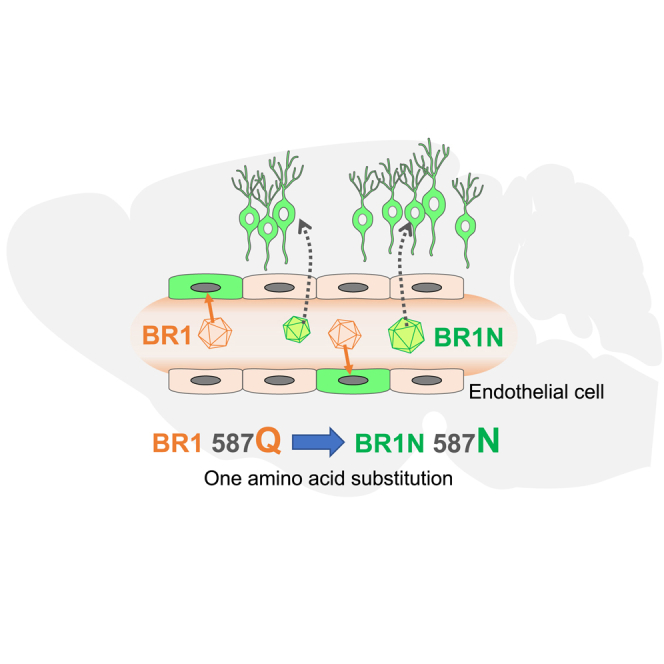

## Teaser

BR1 is an adeno-associated virus serotype 2 (AAV2) capsid variant showing enhanced transduction of brain microvascular endothelial cells in mice following intravenous injection. Kawabata et al. discovered that one amino acid substitution in the BR1 capsid (designated BR1N) makes the capsid highly permeable to the blood-brain barrier.

## Introduction

Adeno-associated viruses (AAVs), which belong to the genus *Dependoparvovirus*, are small, single-stranded, nonenveloped DNA viruses. AAVs infect dividing and quiescent cells, including glial and neuronal cells. Following infection in the absence of a helper virus, the single-stranded AAV genome is converted to double-stranded DNA in the nucleus and stays in the circular episomal form or is preferably integrated into a genomic locus known as *AAVS1* in human cells.^1^ Because AAVs do not cause disease in humans, have broad tissue and cell tropism, and achieve efficient and long-term gene expression with a minimal immune response, AAV-derived vectors are thought to be promising for clinical application as gene therapy vectors.^2^^,^^3^^,^^4^

To date, 13 distinct AAV serotypes (AAV1–AAV13) and more than 100 variants have been isolated from human and non-human primates (NHPs),^5^ with sequence identities among the AAV1–AAV9 capsid proteins ranging from 51% (AAV4 and AAV5) to 99% (AAV1 and AAV6).^6^ The different serotypes have variable tissue and cell tropism.^7^ The canonical AAV2 vector genome can be cross-packaged into the capsid of different serotypes (known as pseudo-serotyping), which enables transduction with broad specificity.^8^ In this study, we used the AAV2 vector genome packaged with different capsids and designated AAV vectors as capsid serotypes (e.g., AAV2/9 as AAV9).

When administered systemically, AAV9 and AAV2 only modestly bind brain microvascular endothelial cells (BMVECs). Upon entering BMVECs, AAV9 is effectively transcytosed, leading to transduction of brain parenchymal cells,^9^^,^^10^ whereas AAV2 is trafficked to the nucleus with little passage through the endothelial barrier, resulting in transduction of BMVECs.^11^^,^^12^ The AAV9 capsid variants AAV-PHP.B and PHP.eB^13^^,^^14^^,^^15^^,^^16^ and the AAV2 capsid mutant BR1^11^^,^^12^ have distinct amino acid insertions in the surface-variable region VIII of their respective capsids, which greatly enhances endocytosis into BMVECs. After entry into BMVECs, they follow intracellular trafficking pathways similar to those of their parent capsids. The AAV9 capsid mutants AAV-PHP.B and PHP.eB cross the endothelial barrier in the original manner of AAV9,^13^^,^^14^^,^^15^^,^^16^ whereas the AAV2 capsid variant BR1 transduces BMVECs identically to AAV2, with minimal transcytosis.^17^

In this study, we show that a single amino acid substitution from glutamine to asparagine at position 587 in the BR1 capsid sequence alters the characteristic that efficiently transduces murine BMVECs into one that effectively crosses the blood-brain barrier (BBB).

## Results

### One amino acid substitution makes the AAV2 capsid mutant BR1 permeable to the BBB

**Figure 1:**
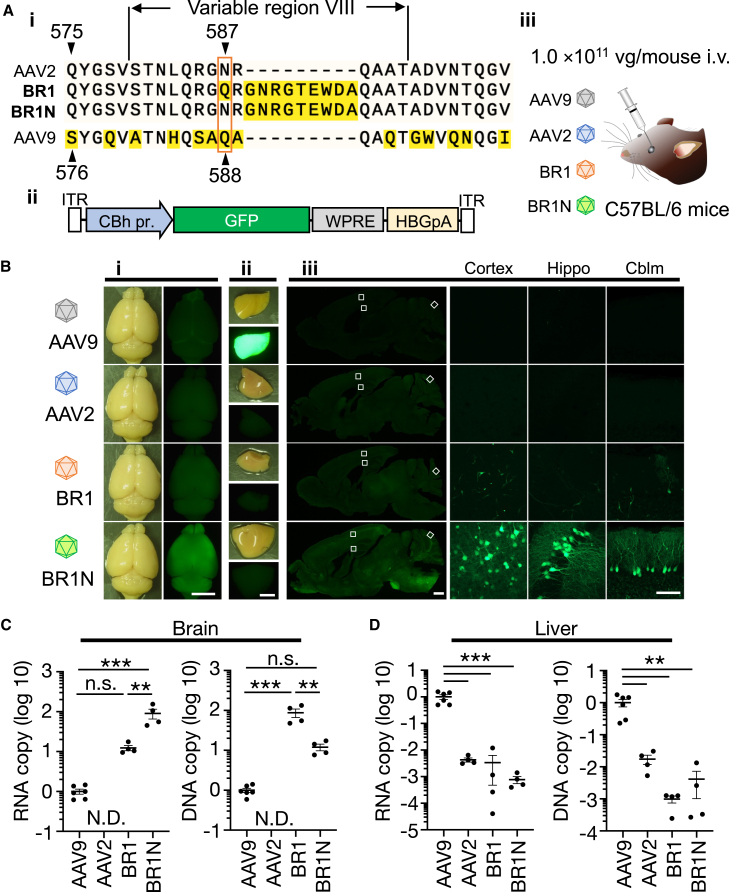
Enhanced CNS tropism of systemically administered BR1N (A-i) Amino acid sequence alignment of AAV capsids (AAV2, BR1, BR1N, and AAV9) around variable region VIII. BR1 and BR1N, AAV2 capsid mutants, have a 7-amino-acid (NRGTEWD) insertion with a “stuffer” sequence (G and A) at both sides in the AAV2 capsid between positions 588 and 589. BR1 also has an asparagine (N)-to-glutamine (Q) substitution at position 587. Amino acids distinct from those of AAV2 are highlighted by a yellow background. (A-ii) Schematic depicting the AAV vector genome. GFP is expressed under control of the CBh promoter. ITR, inverted terminal repeat; WPRE, woodchuck hepatitis virus post-transcriptional regulatory element; HBGpA, human β-globin poly(A). (A-iii) AAV9, AAV2, BR1, or BR1N (1 × 10^11^ vg/mouse, respectively) was injected into adult C57BL/6 mice intravenously (i.v.) through the orbital venous plexus. (B) GFP fluorescence and bright-field images of whole brains (B-i), livers (B-ii), and sagittal brain sections (B-iii) 3 weeks after the AAV injection. Panels in the first to third rows from the right show magnifications of the squares in the cerebral cortex (cortex), hippocampus (Hippo), and cerebellar cortex (Cblm) in the low-power sagittal sections. Scale bars: 5 mm (i and ii), 1 mm (left in iii), and 100 μm (right in iii). (C and D) Logarithmic graphs showing amounts of AAV-derived mRNA and AAV genome. Cortex and liver tissues from female mice treated with AAV9, AAV2, BR1, or BR1N were examined 3 weeks after virus injection. Asterisks indicate statistically significant differences (n = 4–6 mice per group; ∗∗p < 0.01, ∗∗∗p < 0.001 by one-way ANOVA with Bonferroni’s post hoc test for C and D); N.D., not detected; n.s., not significant). All error bars show SEM.

*In vivo* screening of a random AAV2 peptide library was used in a previous study to identify an AAV2 capsid variant enabling specific and efficient transduction of BMVECs upon systemic application.^17^ This mutant, BR1, has heptapeptide (NRGTEWD) insertions with a “stuffer” sequence (G and A) at both sides between amino acid positions R588 and Q589 of the AAV2 capsid protein.^18^ Importantly, BR1 has one more mutation from N to Q at position 587 (Figure 1A-i).^18^ To produce the BR1 capsid for brain microvasculature transduction, we inserted 9 amino acids (7 insertions plus 2 stuffer amino acids) between R588 and Q589 of the AAV2 capsid, where an amino acid at position 587 was still asparagine (termed BR1N). Because we assumed that BR1N might be sufficient for BMVEC transduction, we produced BR1N capsid vectors and intravenously injected them into C57BL/6 mice. However, BR1N transduced cells of the central nervous system (CNS) rather than BMVECs. The results of the preliminary experiment led us to systematically compare the characteristics of BR1N with those of BR1.

We produced AAV9, AAV2, BR1, and BR1N vectors expressing GFP under control of the ubiquitous chicken β-actin hybrid (CBh) promoter (Figure 1A-ii). Each AAV vector (1 × 10^11^ viral genome [vg]/mouse) was injected intravenously into 6- to 10-week-old C57BL/6 mice (Figure 1A-iii). Three weeks after virus injection, GFP expression profiles of the brain were explored. Fluorescence stereomicroscope images showed markedly brighter GFP fluorescence in the whole brain treated with BR1N than in those treated with other AAVs (Figure 1B-i). In contrast, GFP fluorescence in the liver was markedly greater in mice treated with AAV9 than in those treated with wild-type and mutant AAV2 (Figure 1B-ii). Confocal microscopy of sagittal sections of the brain revealed efficient GFP expression in brain parenchymal cells of BR1N-treated mice (Figure 1B-iii). With an increase in injection dose (from 1 × 10^11^ vg/mouse to 3 × 10^11^ vg/mouse or 1 × 10^12^ vg/mouse), GFP expression levels in the whole brain increased in mice systemically injected with BR1N but not in mice treated with AAV9 or BR1 (Figure S1A), whereas GFP fluorescence intensity in the liver was not overtly augmented in mice treated with BR1 and BR1N (Figure S1B).

To quantitatively assess the degree of BBB penetration, we performed quantitative polymerase chain reaction (PCR) measurement of transgene mRNA and viral genome DNA using the brains and livers from mice treated with AAV9, AAV2, BR1, or BR1N (1 × 10^11^ vg/mouse, respectively). Three weeks after systemic infusion of AAVs, tissues were harvested and homogenized, followed by purification of viral DNA and mRNA. Quantitative reverse-transcriptase PCR (RT-PCR) and real-time PCR revealed 90-fold and 12-fold increases in transgene mRNA levels and viral DNA content, respectively, in the brain treated with BR1N compared with brains treated with AAV9 (Figure 1C). The amounts of transgene mRNA and viral DNA in the AAV2 treated brain were under the detection limit (Figure 1C). Similarly, we measured AAV-derived mRNA and DNA in the liver. Consistent with the GFP fluorescence profile in the liver (Figure 1B-ii), GFP mRNA and viral DNA content in the liver treated with AAV2 or BR1N were significantly lower than in those treated with AAV9 (Figure 1D). There were no significant differences in the amounts of transgene mRNA and viral DNA in the liver between AAV2-treated mice and those treated with BR1N.

### Significantly higher CNS tropism of BR1N than BR1

**Figure 2:**
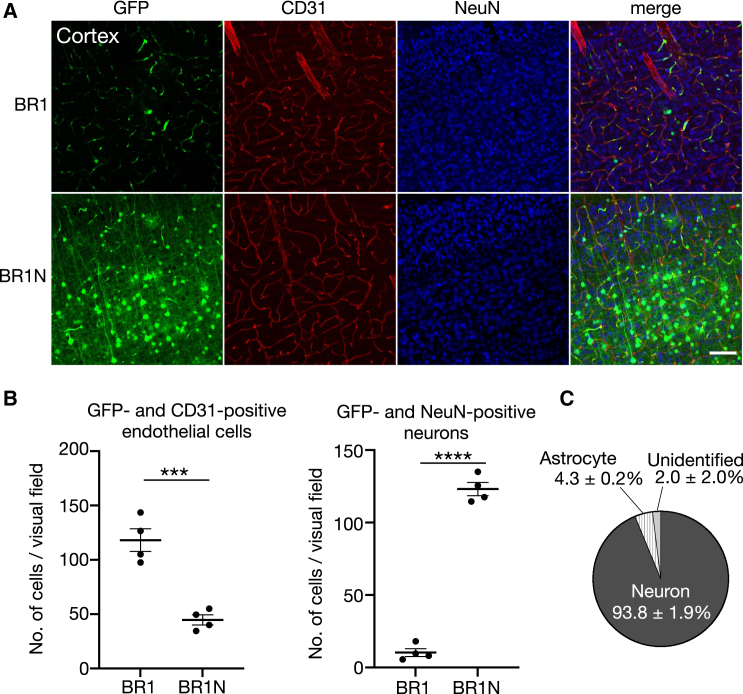
Enhanced neuron transduction by i.v. injected BR1N (A) Immunohistochemistry of brain sagittal sections from male mice treated i.v. with BR1 or BR1N. The sagittal sections were double-immunolabeled for CD31, a microvasculature endothelial cell marker, and NeuN, a neuronal marker. Scale bar, 100 μm. (B) Graphs showing the number of cells double-positive for GFP and CD31 (transduced endothelial cells, left graph) and those double-positive for GFP and NeuN (transduced neurons, right graph). Asterisks show statistically significant differences (n = 4 mice per group; ∗∗∗p < 0.001 and ∗∗∗∗p < 0.0001 by unpaired t test). (C) Percent ratio of transduced cell types to total brain parenchymal cells (GFP-positive and CD31-negative cells) (n = 4 mice). Cerebral sections were immunolabeled for CD31, NeuN, the astrocyte marker S100, or the microglia marker Iba1. All error bars show SEM.

Brain sections from BR1- and BR1N-treated mice were double-immunolabeled for CD31, a microvasculature endothelial cell marker, and NeuN, a neuronal marker. Confocal microscopy showed numerous cells double-labeled for GFP and CD31 in the BR1-treated brain, indicating efficient transduction of brain endothelial cells (Figure 2A, top panels), consistent with a previous study.^17^ In contrast, BR1N-treated brains showed many GFP and NeuN double-positive cells (Figure 2A, bottom panels). Quantitative analysis revealed significantly higher numbers of transduced endothelial cells in BR1-treated brain (118 ± 9 cells/0.408 mm^2^ visual field, n = 4 mice) than in BR1N-treated brain (45 ± 4 cells/visual field, n = 4 mice), whereas significantly higher numbers of transduced neurons were observed in BR1N-treated brain (123 ± 4 cells/visual field, n = 4 mice) than in BR1-treated brain (10 ± 2 cells/visual field, n = 4 mice) (Figure 2B). To identify types of GFP-positive and CD31-negative cells (i.e., brain parenchymal cells) in BR1N-treated brain, the sections were immunolabeled for NeuN, an astrocyte marker (S100), or a microglia marker (Iba1) together with CD31. We assessed a total of 1,410 GFP-positive and CD31-negative cells in 8 visual fields from 4 mice. The results showed that 94% of GFP-expressing cells in BR1N-treated brain were NeuN-positive neurons, with the rest of the cells being astrocytes (4%) and unidentified cells (2%) (Figures 2C and S2A). We found no GFP-positive cells co-labeled for Iba1 (Figure S2B). These results suggest that endocytosed BR1 was trafficked to the nucleus and transduced the BMVECs, whereas the majority of endocytosed BR1N was transcytosed through the endothelial cells and principally transduced neurons in the C57BL/6 mouse brain.

### Comparison of the CNS tropism of BR1N with that of PHP.eB

**Figure 3:**
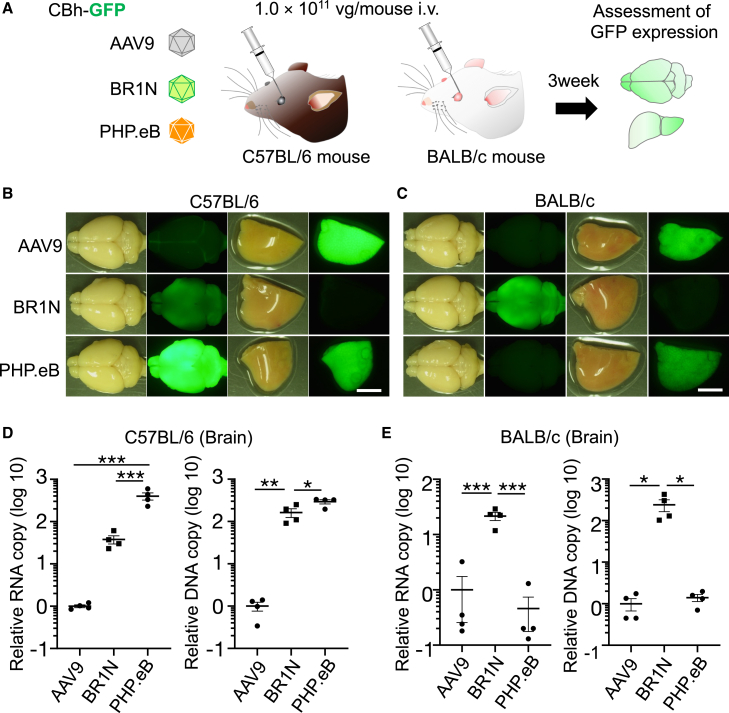
Efficient CNS transduction in BALB/c mice by i.v. injected BR1N (A) AAV9, BR1N, or PHP.eB expressing GFP under control of the CBh promoter (1 × 10^11^ vg/mouse) was injected i.v. into C57BL/6 mice or BALB/c mice. Three weeks after the AAV injection, GFP expression was examined in the brain and liver. (B and C) Representative GFP fluorescence images of the whole brain and liver from AAV-treated C57BL/6 (B) and BALB/c (C) mice. Scale bars, 5 mm. (D and E) Logarithmic graphs showing the amounts of AAV-derived mRNA and viral DNA copy numbers in cerebral hemispheres from C57BL/6 (D) and BALB/c (E) mice treated with AAV9, BR1N, or PHP.eB. Data were obtained by qRT-PCR and real-time PCR 3 weeks after virus injection. Asterisks indicate statistically significant differences (n = 4 mice per group; ∗p < 0.05, ∗∗p < 0.01, and ∗∗∗p < 0.001 by one-way ANOVA with Bonferroni’s post hoc test). All error bars show SEM.

PHP.eB, an AAV9 capsid variant with a 7-amino-acid (TLAVPFK) insertion and flanking 2-amino-acid mutation at variable region VIII,^13^ was shown to cross the BBB with markedly higher efficacy than the parent AAV9.^13^ The enhanced CNS tropism of PHP.eB was limited to C57BL/6 and phylogenetically close mouse strains and not observed in distant mouse strains such as BALB/c mice.^15^^,^^16^^,^^19^ This is because PHP.eB uses LY6A (glycosylphosphatidylinositol [GPI]-linked lymphocyte antigen 6 complex, locus A), which exists in the luminal membrane of BMVECs, as the receptor for crossing the endothelial barrier; inbred strains expressing LY6A with intact GPI anchoring and membrane localization show higher CNS tropism than the parent AAV9, whereas the capacity to cross the BBB in mouse strains with GPI-anchoring-disrupted *Ly6a*, such as the BALB/c strain, remains as low as in AAV9.^15^^,^^16^^,^^20^ If BR1N uses LY6A as a receptor for crossing the BBB, then intravenously administered BR1N likely fails to cross the BBB in BALB/c mice, similar to PHP.eB. To verify this, we injected BR1N, PHP.eB, or AAV9 (1.0 × 10^11^ vg/mouse) intravenously into BALB/c mice and C57BL/6 mice (as a control) and compared the CNS tropism 3 weeks after virus injection (Figure 3A).

In C57BL/6 mice, PHP.eB caused the highest brain transduction, followed by BR1N and AAV9, whereas marked liver transduction was seen in mice treated with AAV9 and its capsid variant (AAV-PHP.eB) (Figure 3B). GFP in the liver was hardly detectable in C57BL/6 mice treated with BR1N (Figure 3B). In BALB/c mice, AAV9 and AAV-PHP.eB transduced the liver with high efficacy but failed to transduce the CNS (Figure 3C, top and bottom panels). In sharp contrast, BR1N caused efficient brain transduction and little liver transduction (Figure 3C, center panels).

The transduction profiles of the brain after the systemic application of the respective AAVs were confirmed by quantitative PCR. The amount of AAV-derived mRNA and that of viral genome DNA in the brain were highest in C57BL/6 mice treated with PHP.eB, followed by those with BR1N and lowest in those with AAV9 (Figure 3D). In BALB/c mice, the transgene mRNA and viral genome DNA were significantly higher in brains treated with BR1N than in those treated with AAV9 or PHP.eB (Figure 3E). There were no statistically significant differences in transgene mRNA and viral genome DNA between BALB/c mouse brains treated with AAV9 and those treated with PHP.eB.

### No cross-immunity between BR1N and PHP.eB

**Figure 4:**
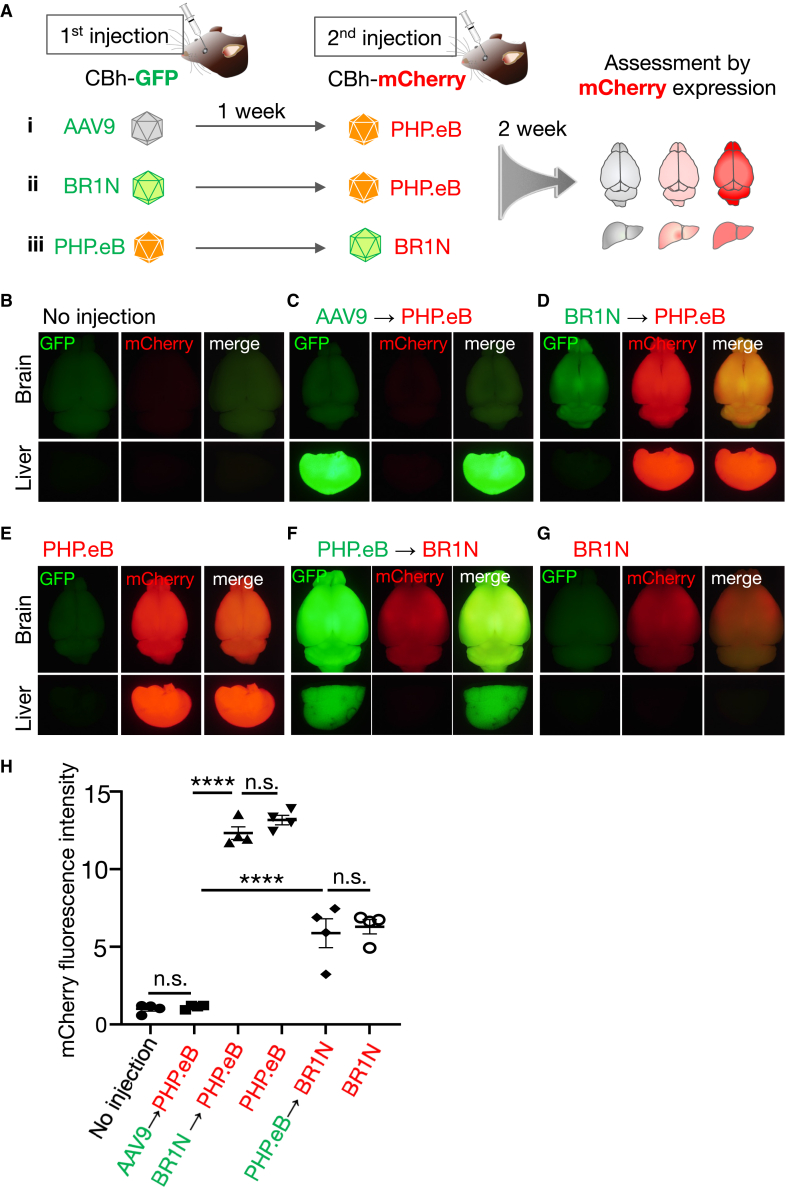
No cross-reaction of NAbs between AAV9 and BR1N (A-i–A-iii) C57BL/6 mice received systemic AAV injections twice with a 1-week interval. The first injection (AAVs expressing GFP, 1 × 10^11^ vg/mouse) aimed to produce NAbs, whereas the second injection (2 × 10^11^ vg/mouse) used different capsid AAVs expressing mCherry to test the cross-reactivity of the NAbs produced by the first AAV injection. Cross-reactivity was assessed by mCherry fluorescence intensity. (B–G) Representative GFP and mCherry fluorescence images of the whole brain and liver from mice treated as described above. Fluorescence images of whole brains and livers from naive mice (B) and mice treated with mCherry-expressing PHP.eB alone (E) or mCherry-expressing BR1N alone (G) are presented as controls. (H) Summarized graph showing the mCherry fluorescence intensity from whole brains. Asterisks indicate statistically significant differences (n = 4 mice per group; ∗∗∗∗p < 0.0001 by one-way ANOVA with Bonferroni’s post hoc test). All error bars show SEM.

Our previous study using mice showed production of neutralizing antibodies (NAbs) against the AAV-PHP.B capsid 7 days after the systemic infusion.^21^ Accordingly, the second systemic application of AAV-PHP.B, 7 days after the first systemic AAV-PHP.B injection, failed to transduce the brain. The AAV2 capsid has 81% amino acid sequence homology with that of AAV9;^6^ however, NAbs against BR1N (the AAV2 capsid mutant) may not cross-react with PHP.eB (the AAV9 capsid variant). To test this, we intravenously injected AAV9 or BR1N, which expresses GFP under control of the CBh promoter, into C57BL/6 mice. One week after the first injection, a second systemic infusion of PHP.eB expressing mCherry under control of the CBh promoter was performed (Figure 4A-i and -ii). Two weeks after the second injection, transduction profiles of the brain and liver were examined (Figure 4A, right diagram).

The first AAV9 injection resulted in an absence of expression of the second transgene (mCherry) by PHP.eB in the brain and liver (Figure 4C), confirming a previous study.^21^ In contrast, the first BR1N injection did not disturb expression of the second transgene (mCherry) by PHP.eB in the brain and liver (Figure 4D). Next, we reversed the injection order (PHP.eB-GFP → BR1N-mCherry) (Figure 4A-iii). Again, the first PHP.eB injection did not interfere with second-BR1N-mediated mCherry expression in the brain (Figure 4F). Although mCherry expression in the liver by BR1N was not clearly observed (Figure 4F), this was likely due to low liver tropism of BR1N, analogous to AAV2. Quantitative analysis of mCherry fluorescence intensity in the whole brain 2 weeks after the second virus injection confirmed the absence of cross-reactivity of NAbs between BR1N and PHP.eB (Figure 4H). Results consistent with this were also observed in sagittal brain sections (Figure S3).

Generation of cross-reacting antibodies against the AAV2 variant, BR1N, after pretreatment with a different AAV9 capsid variant, PHP.eB, or vice versa may require active class switch recombination (CSR) and/or somatic hypermutation (SHM).^22^ Because 1 week between injections may not be enough to allow those changes in adaptive immunity, we repeated similar experiments with a 4-week interval between the first and second injection (Figure S4A). We confirmed that mCherry expression by PHP.eB (or BR1N) was not significantly influenced 4 weeks prior to injection of BR1N (or PHP.eB) (Figures S4B–S4F). These results suggest that the NAbs against BR1N do not cross-react with PHP.eB and vice versa.

### Efficient BBB penetration of the AAV with a mosaic capsid composed of BR1 and AAV9

AAV9 and AAV2 have only a modest binding capacity to the luminal membrane of BMVECs. However, when they are endocytosed, AAV9 is sorted to a route crossing the endothelial barrier, whereas AAV2 is trafficked to the nucleus and transduces BMVECs.^12^^,^^23^ PHP.B and BR1 are thought to have higher membrane binding and, consequently, enhanced endocytosis, than their original AAVs, but upon endocytosis, they are trafficked along similar routes as their parent AAVs. Thus, it is intriguing to examine the transduction profile of a mosaic capsid AAV^24^ composed of the BR1 mutant (enhanced endocytosis into BMVECs and trafficking to the nucleus) and wild-type AAV9 (low endocytosis but efficient passage through BMVECs).

**Figure 5:**
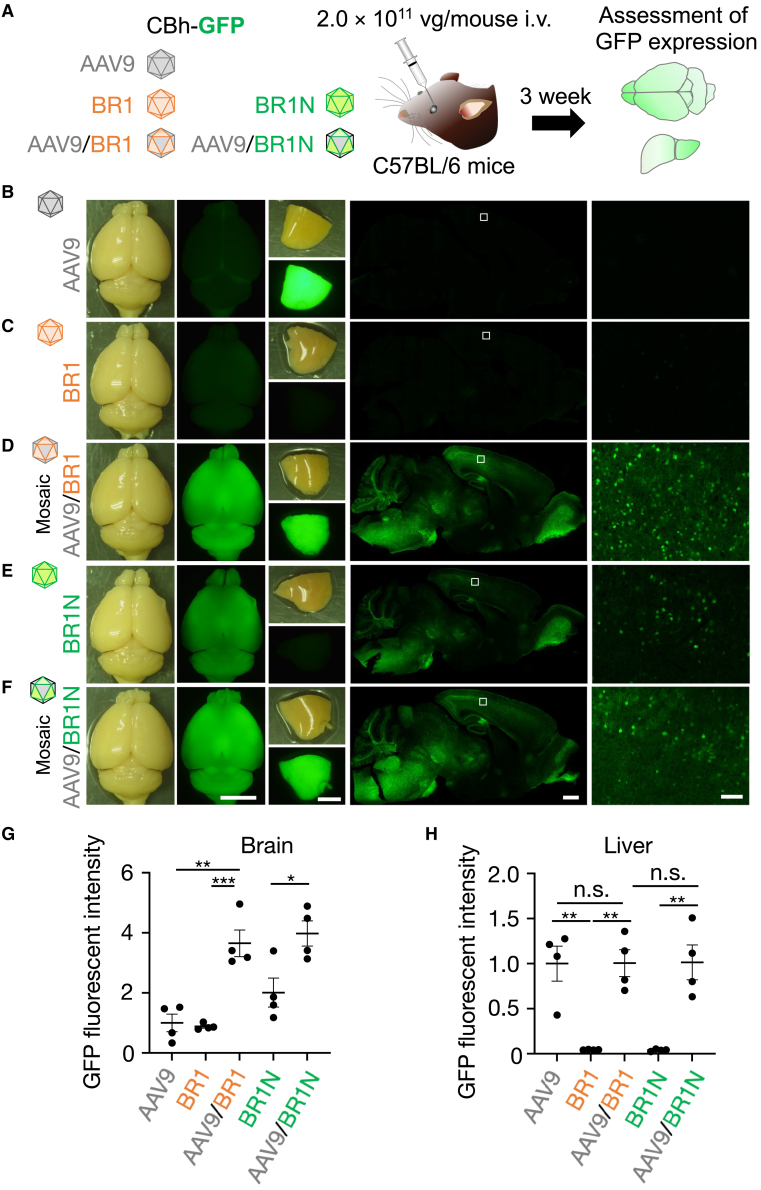
Efficient brain transduction by AAVs with a mosaic capsid composed of AAV9 and BR1 (A) Diagram showing experimental procedures. C57BL/6 mice received i.v. injections of uniform or mosaic capsid AAVs expressing GFP under control of the CBh promoter (2.0 × 10^11^ vg/mouse, respectively). Three weeks after virus injection, GFP expression profiles of the brain and liver were examined. (B–D) Bright-field and GFP fluorescence images of brains and livers from mice treated with AAV9 (B), BR1 (C), the AAV9/BR1 mosaic capsid vector (D), BR1N (E), and the AAV9/BR1N mosaic capsid vector (F). Panels in the second row from the right show GFP fluorescence images of sagittal whole-brain sections, from which square regions in the cortex are magnified and presented on the right. Scale bars, 5 mm for whole brains and livers and 1 mm (left) and 100 μm (right) for brain sections. (G, H) Summarized graph showing GFP fluorescence intensity from whole brains (G) and livers (H). Asterisks indicate statistically significant differences (n = 4 mice per group; ∗p < 0.05, ∗∗p < 0.01, and ∗∗∗p < 0.001 by one-way ANOVA with Bonferroni’s post hoc test). All error bars show SEM.

We produced mosaic AAV vectors (AAV9/BR1) whose capsid was composed of AAV2 mutant BR1 and wild-type AAV9 using a 1:1 mixture of the *rep*/*cap* plasmids expressing the respective capsid proteins. GFP-expressing AAV9, BR1, or mosaic AAV9/BR1 was injected intravenously into 6- to 10-week-old C57/BL6 mice (Figure 5A). Three weeks after the injection, the native GFP expression profiles of the whole brain and sagittal brain sections were examined. Fluorescence stereoscopy and microscopy showed only modest GFP expression in the brains from AAV9- or BR1-treated mice and conspicuous GFP expression in AAV9-treated mouse livers (Figures 5B and 5C). In contrast, mice treated with the AAV9/BR1 mosaic capsid vector showed marked GFP expression in the brain as well as the liver (Figure 5D). We did a similar experiment using a mosaic capsid AAV composed of the AAV9 capsid and BR1N (AAV9/BR1N) in place of BR1. Again, we found enhanced GFP expression in the brain and liver in mice treated with AAV9/BR1N compared with mice treated with BR1N (Figures 5E and 5F). Quantitative analysis confirmed significantly greater CNS tropism of mosaic AAV9/BR1 and AAV9/BR1N than of uniform AAV9, BR1, and BR1N (Figure 5G). Transduction of the liver by mosaic AAV9/BR1 and AAV9/BR1N was significantly greater than by uniform BR1 and BR1N and comparable with that by AAV9 (Figure 5H).

### Little transduction of the marmoset brain after intravenous infusion of BR1N

Using common marmosets, we tested whether intravenously administered BR1N also penetrated the BBB and transduced the brain in non-human primates. Male marmosets 1.4 years old received an intravenous infusion of BR1N or AAV9 (5 × 10^11^ vg/kg), and the brains were examined 4 weeks after the treatment. A sagittal brain section from an AAV9-treated marmoset showed GFP-positive cells scattered throughout the brain, whereas that from a BR1N-treated marmoset showed a markedly lower population of GFP-positive cells (Figure S5). Quantification of viral DNA from the cerebral cortex revealed approximately one-fourth of the DNA amount in the BR1N-treated brain compared with that in the AAV9-treated brain. Notably, there were almost no GFP-labeled BMVECs in BR1N-treated brain. These results suggest that, unlike in mice, entry of BR1N into BMVECs was not enhanced in marmosets, and, thus, intravenously infused BR1N behaves essentially as the parent AAV2 in marmosets.

## Discussion

BR1 is an AAV2 capsid variant showing enhanced transduction of BMVECs in mice following intravenous injection.^17^ In the present study, we showed that one amino acid substitution from glutamine to asparagine at position 587 in the surface-variable region VIII of the BR1 capsid converted the characteristic that efficiently transduces murine BMVECs into one that effectively crosses the BBB, with liver transduction similarly low as that by the parent AAV2. The BBB penetration capacity of BR1N is significantly higher than that of AAV9; although systemic administration of BR1N still transduces BMVECs, the vast majority of transduced cells were brain parenchymal cells, with over 90% of the cells being neurons.

Upon entry into BMVECs, AAV2 is trafficked to the nucleus and transduces the cells, whereas AAV9 and AAVrh10 are directed to the transcytosis pathway,^12^^,^^25^^,^^26^ suggesting that a range of capsid sequences likely enables BBB crossing. Although the intracellular sorting mechanism remains unknown, it is speculated that some protein bound to the AAV9, AAVrh10, or AAV2 capsid determines the intracellular routes for transduction or transcytosis. Therefore, comparison of proteins bound to AAV9 or AAVrh10 with those bound to AAV2 may help to identify key molecules that determine transduction or transcytosis in BMVECs. However, because the amino acid sequence of capsids differs by approximately 18% between AAV2 and AAV9^6^ and 16% between AAV2 and AAVrh10, there may be numerous candidates found.

BR1, which has an arginine-to-glutamine mutation at position 587, transduced BMVECs more efficiently than AAV2 and BR1N, both of which contain arginine at position 587. Therefore, in addition to the natural trend of AAV2 toward transduction over transcytosis, introduction of the N587Q mutation in BR1 may generate a backbone that globally impairs vascular transcytosis. Thus, comparison of proteins bound to BR1 with those bound to one amino acid-distinct BR1N is likely more effective for identification of a critical molecule that guides endocytosed viral particles toward transduction or transcytosis.

Upon systemic injection, PHP.B and PHP.eB showed higher BBB penetration efficiency than their parent AAV9 in C57BL/6 mice but not in BALB/c mice.^15^^,^^16^^,^^19^ This is because LY6A, the receptor localizing on the luminal membrane of BMVECs and responsible for the enhanced CNS tropism of PHP.B and PHP.eB, is mutated in BALB/c mice, which disrupts the GPI anchoring and membrane localization of LY6A.^16^ Our result that intravenous infusion of BR1N showed enhanced CNS tropism in the BALB/c strain as well as in the C57BL/6 strain suggests a receptor distinct from LY6A for BR1N binding to BMVECs. BR1 and BR1N probably use the same receptor, and yet a minor sequence modification (one amino acid) has profound consequences on tropism quantitatively and qualitatively. This is potentially a very important paradigm in capsid design, showing that receptor binding alone does not determine the final outcome *in vivo*. This finding suggests that further improvements of capsids within a predetermined receptor usage are feasible.

Systemic application of PHP.eB and BR1N at 1- and 4-week intervals (Figures 4 and S4) showed that NAbs against PHP.eB did not cross-react with BR1N and vice versa. These results suggest that two different transgenes can be expressed in the mouse CNS by systemic administration after a certain period. For example, after a disease model mouse is produced by PHP.eB-mediated expression or knockdown of a disease-causing gene in the CNS, as reported previously,^27^^,^^28^ therapeutic efficacy can be explored by systemically delivering a therapeutic gene using BR1N to the CNS of the virally generated model mouse. However, the CNS tropism of BR1N, which was assessed by expression levels of transgene mRNA in the brain tissue, still remained one 10th of that of PHP.eB (Figure 3D). Thus, development of an AAV2 capsid variant that transduces the brain more effectively than BR1N may be required to obtain substantial therapeutic efficacy.

Our capsid mosaicism experiments transfected cells with the two different packaging (*rep*/*cap*) plasmids at a 1:1 ratio, which generates a mix of different mosaic and “parental” capsids. Notably, use of very low amounts of packaging plasmids results in production of mostly “parental” capsid AAVs, and the ratio of mosaic capsid vectors to total AAV vectors increases with the amount of *rep*/*cap* plasmids.^29^ We used 30 μg *rep*/*cap* plasmid per 24.5 cm, which is close to a saturating condition and is thought to generate a substantial number of mosaic vectors but still contain partly uniform capsid vectors.

BBB-permeable capsid variants with less accumulation in the liver, such as BR1N, are valuable for mitigating liver toxicity because liver damage is a significant problem with systemic administration of AAV9.^30^^,^^31^ Unfortunately, intravenously infused BR1N failed to cross the marmoset BBB, probably because BR1N could not be effectively entrapped by BMVECs in marmosets. A similar result was observed for PHP.B, which showed enhanced BBB penetration in mice but not in marmosets^10^ because of the absence of LY6A, a binding partner for PHP.B, in marmosets.^15^^,^^16^ Nevertheless, the discovery of PHP.B facilitated subsequent challenges for screening the AAV9 capsid library in marmosets, leading to recent successful identification of AAV9 capsid variants with enhanced BBB penetration.^32^^,^^33^ Thus, the present results may open a new avenue for development of capsid variants from diverse AAV serotypes that effectively cross the BBB in NHPs and humans.

## Materials and methods

### AAV vector preparation

The expression plasmid pAAV is comprised of the CBh promoter, GFP, woodchuck hepatitis virus post-transcriptional regulatory element (WPRE), and the human β-globin polyadenylation signal sequence;^21^ GFP was inserted into the AgeI/NotI-digested site of the pAAV-CBh plasmid. The packaging plasmids for BR1 and BR1N were constructed by replacing a BsiWI/NdeI fragment in pRC2-mi342 with respective mutant capsid gene fragments containing the additional peptide sequence. Recombinant single-stranded AAV vectors were produced by the ultracentrifugation method as reported previously,^34^ except for those used in Figure 1. Briefly, three plasmids, the expression plasmid (pAAV/CBh-GFP-WPRE-HBGpA), pHelper (Agilent Technologies, Santa Clara, CA, USA), and the packaging plasmid (pAAV2, pAAV9, pBR1, or pBR1N), were co-transfected using polyethylenimine into HEK293T cells (HCL4517; Thermo Fisher Scientific, Waltham, MA, USA) cultured in Dulbecco’s modified Eagle’s medium (DMEM; D5796-500Ml; Sigma-Aldrich, St. Louis, MO, USA) supplemented with 8% fetal bovine serum (Sigma-Aldrich). For production of mosaic vectors (AAV9/BR1 and AAV9/BR1N), the packaging plasmids pAAV9 and pBR1/BR1N were used at a 1:1 ratio. Viral particles were harvested from the culture medium 6 days after transfection and concentrated by precipitation with 8% polyethylene glycol 8000 (P5413; Sigma-Aldrich) and 500 mM sodium chloride. The precipitated AAV particles were resuspended in Dulbecco’s phosphate-buffered saline (D-PBS) and purified with iodixanol (Optiprep; AXS-1114542-250ML; Alere Technologies, Oslo, Norway) step gradient ultracentrifugation. The viral solution was further concentrated and formulated with D-PBS using a Vivaspin 20 column (VS2041 or VS2042; Sartorius, Göttingen, Germany). AAV vectors used in Figure 1 (AAV2, AAV9, BR1, and BR1N) were produced by the modified minimally purified method,^34^ which differs from the conventional ultracentrifugation method in the harvesting protocol: viral particles were collected 4 days after transfection from the culture medium and the producing cells. HEK293T cells received 3 freeze-thaw cycles to release the viral particles. The cell debris was removed using a syringe filter, and the viral solution was further concentrated and formulated with D-PBS using a Vivaspin 20 column. The genomic titers of the viral vector were determined by quantitative real-time PCR using Power SYBR Green PCR Master Mix (Thermo Fisher Scientific) and primers 5′-CTGTTGGGCACTGACAATTC-3′ and 5′-GAAGGGACGTAGCAGAAGGA-3′ for the WPRE sequence. The expression plasmid was used as a standard.

### Animals

Wild-type mice from C57BL/6J and BALB/c backgrounds, purchased from Charles River Laboratories Japan (Yokohama, Japan) and Japan SLC (Hamamatsu, Japan), respectively, were used in this study. All procedures for the care and treatment of animals were performed according to the Japanese Act on the Welfare and Management of Animals and the Guidelines for Proper Conduct of Animal Experiments issued by the Science Council of Japan. Common marmosets (*Callithrix jacchus*) were maintained in breeding rooms under controlled conditions (temperature, 26°C–28°C; humidity, 20%–60%; 12-h dark/light cycle with light turning on at 7 a.m.) as described previously.^35^ Filtered water was provided *ad libitum*, and 40–50 g of soaked monkey chow (CMS-1, CLEA, Japan) supplemented with vitamins and fruits, vegetables, boiled chicken, milk powder, or powder of *Lactobacillus* was provided daily. Two marmosets were kept in a large cage (750 × 550 × 762 mm) equipped with peach tree branches and poplar wood perches. All animals were handled according to the Guide for the Care and Use of Laboratory Animals, 8th edition. The experimental protocols were approved by the Institutional Committee of Gunma University (21-063 and 21-065). All efforts were made to minimize suffering and to reduce the number of animals used.

### Intravenous injection of AAVs into the mouse orbital venous plexus

After deep anesthesia via an intra-peritoneal injection of ketamine (100 mg/kg body weight) and xylazine (10 mg/kg body weight), 100 μL of the AAV vector preparation was intravenously injected into the retro-orbital sinus of mice using a 1-mL syringe with a 30G needle (08277, Nipro, Osaka, Japan) for 20–30 s.

### Intravenous injection of AAVs into the marmoset femoral vein

Marmosets were lightly anesthetized with ketamine (10 mg/kg) and xylazine (0.8 mg/kg). AAV vectors in a 1-mL syringe were injected into the femoral vein through a 27G winged needle with a silicon tube (SV-270DL, Terumo, Tokyo, Japan) that was connected to a three-way stopcock Planecta (JV-PNSC1B, JMS, Hiroshima, Japan). The viral solution remaining in the silicon tube was flushed out by an additional injection of physiological saline solution (Otsuka Pharmaceutical Factory, Tokushima, Japan).

### Quantification of the amount of AAV-derived mRNA and AAV genome DNA in mice

AAV-derived mRNA was isolated from a cerebral hemisphere using TRIzol lysis reagent (Thermo Fisher Scientific) or QIAzol lysis reagent (QIAGEN, MD, USA). Reverse transcription was performed using ReverTra Ace quantitative RT-PCR Master Mix with gDNA Remover (Toyobo, Osaka, Japan) to synthesize cDNA from the extracted mRNA samples, and quantitative PCR was performed using Power SYBR Green PCR Master Mix (Thermo Fisher Scientific). The forward (F) and reverse (R) primers had the following sequences: Gapdh-F, 5′-ACAACTTTGGCATTGTGGAA-3′; Gapdh-R, 5′-GATGCAGGGATGATGTTCTG-3′; GFP-F, 5′-CGACCACTACCAGCAGAACAC-3′; GFP-R, 5′-TGTGATCGCGCTTCTCGTTGG-3′.

To quantify the amount of viral DNA, we collected the RNA phase, which contained the viral genome, from TRIzol- or QIAzol-treated tissue samples. After digestion of unnecessary RNA by treatment with RNaseA (Fujifilm, Osaka, Japan), quantitative PCR was performed using Power SYBR Green PCR Master Mix (Thermo Fisher Scientific). The F and R primers used had the following sequences: GFP-F, 5′-CGACCACTACCAGCAGAACAC-3′; GFP-R, 5′-TGTGATCGCGCTTCTCGTTGG-3′; Rosa26-F, 5′-AAGGCTAACCTGGTGTGTGG-3′; Rosa26-R, 5′-GGCGGATCACAAGCAATAAT-3′. The thermal cycling program used had an initial denaturation step of 95°C for 10 min, followed by 40 cycles at 95°C for 15 s and 60°C for 60 s.

### Quantification of the amount of AAV genome DNA in marmosets

AAV genome DNA was isolated from the cerebral cortex of marmosets using the Wizard Genomic DNA Purification Kit (Promega, Madison, WI, USA). Quantitative PCR was performed using Power SYBR Green PCR Master Mix (Thermo Fisher Scientific). The F and R primers had the following sequences: cjGAPDH-F, 5′-GTGTGTCAAATGTTTCCTGGG-3′; cjGAPDH-R, 5′-CTCAGTTTCCTCCTCCGTAAG-3′; GFP-WPRE-F: 5′-GGACGAGCTGTACAAGTAAAG-3′; GFP-WPRE-R: 5′-GGGAAGCAATAGCATGATACAAAGG-3′. The thermal cycling program used had an initial denaturation step at 95°C for 10 min, followed by 40 cycles at 95°C for 15 s and then 60°C for 60 s.

### Immunohistochemistry

Between 20 and 22 days after virus injection, deeply sedated mice were transcardially perfused with PBS (pH 7.4) and 4% paraformaldehyde in 0.1 M phosphate buffer (4% PFA/PB). The whole brain was immersed in 4% PFA/PB for 4–5 h at 4°C and cut into 50-μm sagittal sections using a vibratome (VT1200 S, Leica Microsystems, Wetzlar, Germany). Free-floating sagittal brain sections were blocked with PB containing 2% normal donkey serum, 2% BSA, 0.05% Triton X-100, and 0.05% NaN_3_ (blocking solution), and then they were incubated overnight at room temperature (24°C–26°C) with primary antibodies. The primary antibodies were rat monoclonal anti-CD31 (1:100, 550274, BD Pharmingen, NJ, USA), mouse monoclonal anti-NeuN (1:1,000, MAB377, Merck, Darmstadt, Germany), rabbit polyclonal anti-S100β (1:1,000, S100β-Rb-Af1000, Frontier Institute, Hokkaido, Japan), and rabbit polyclonal anti-Iba1 (1:500, 019-19741, Fujifilm Wako, Osaka, Japan). After rinsing several times with PBS containing Triton X-100 at room temperature, the slices were incubated with the relevant secondary antibodies for 3 h at room temperature in blocking solution containing the following secondary antibodies: Alexa Fluor Plus 647 donkey anti-rat immunoglobulin G (IgG; 1:2,000) and Alexa Fluor Plus 555 donkey anti-mouse or anti-rabbit IgG (1:2,000). After washing using the same procedure as above, immunostained sections were mounted on glass slides with ProLong Diamond Antifade reagent (Thermo Fisher Scientific).

### Imaging analysis

The fluorescence images of brain sections in Figures 2, S2, S5-i, and S5-ii were acquired using a laser-scanning confocal microscope (LSM 800, Carl Zeiss, Oberkochen, Germany) with a 10× objective, and z stack images of different focal planes were generated. GFP (+) and immunolabeled cells were counted manually. Fluorescence images of sagittal whole-brain sections and the enlarged images were acquired with a fluorescence microscope (BZ-X700 or BZ-X-800, Keyence, Osaka, Japan) with 10× and 20× objectives, respectively. GFP fluorescence images of the whole brain and liver were acquired using a fluorescence stereoscopic microscope (VB-7010, Keyence, Osaka, Japan). The GFP fluorescence intensity of the whole brain and liver was measured using ImageJ software. The outline of the whole brain or liver was traced, and the fluorescence intensity in the enclosed areas was measured accordingly.

### Statistical analysis

GraphPad Prism v.9 (GraphPad, San Diego, CA, USA) was used for statistical analysis and production of graphic images. Because of ethical concerns, we tried to use as few animals as possible, and our data came from small samples. Therefore, we first checked the normality of our data using a quantile-quantile plot.^36^ After validation of the normal distribution, we used one-way ANOVA with Bonferroni’s post hoc test or unpaired t test for statistical analysis. Statistical methods used are indicated in each figure legend. The data are expressed as mean ± standard error (SEM), and p < 0.05 was considered statistically significant.

## Data Availability

Packaging plasmids for any of the new capsids described herein are available through a material transfer agreement (MTA) with Gunma University.

## Supplemental information

Document S1. Figures S1–S5

Document S2. Article plus supplemental information
