# Supplementary material for: A blood-brain barrier-penetrating AAV2 mutant created by a brain microvasculature endothelial cell-targeted AAV2 variant
Source: Mol Ther Methods Clin Dev. 2023 Mar 2;29:81–92. doi: 10.1016/j.omtm.2023.02.016 (PMC10036519; doi:10.1016/j.omtm.2023.02.016)
Supplement: Document S1. Figures S1–S5 [file mmc1.pdf]

OMTM, Volume *29*

Supplemental information

A blood-brain barrier-penetrating AAV2 mutant created by a brain microvasculature endothelial cell-targeted AAV2 variant

Hayato Kawabata, Ayumu Konno, Yasunori Matsuzaki, and Hirokazu Hirai

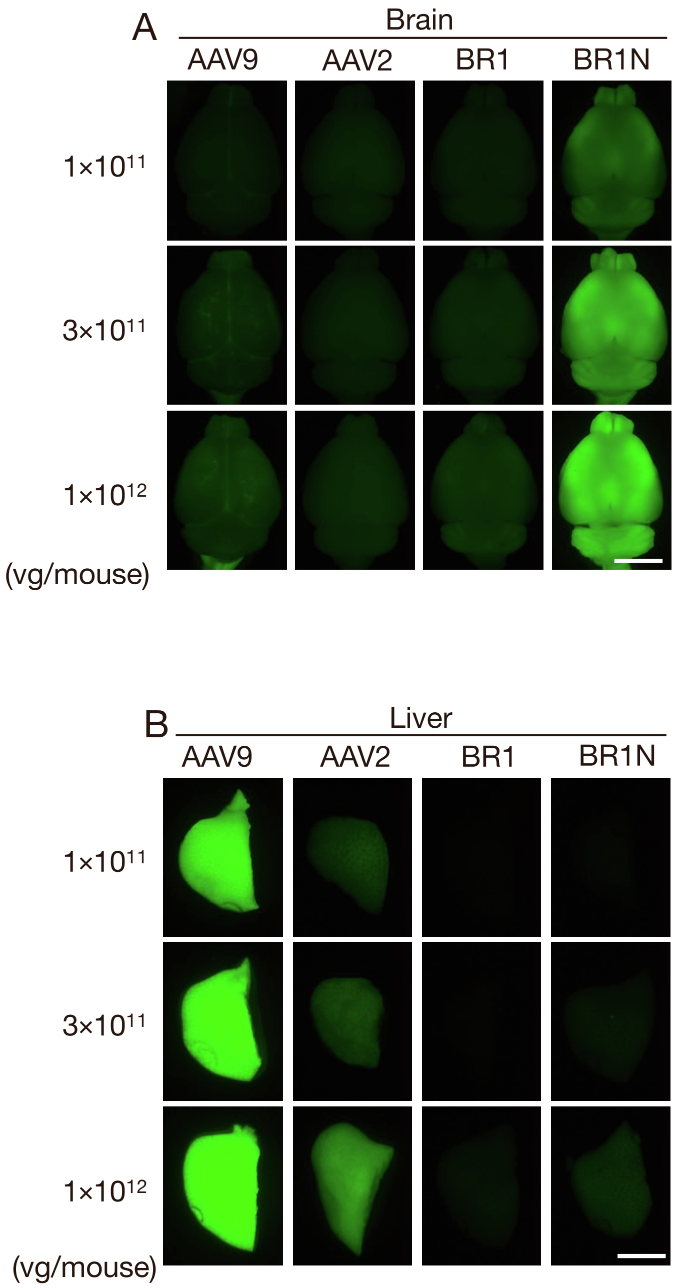

Figure S1. Injection dose-dependent CNS tropism of BR1N. C57BL/6 mice received intravenous injection of AAV9, BR1, or BR1N expressing GFP under the control of the CBh promoter at a dose of 1×10^{11}, 3×10^{11}, or 1×10^{12} vg/mouse. Three weeks after the AAV injection, GFP expression was examined in the brain (**A**) and liver (**B**). Note the elevation of the GFP fluorescence in BR1N-treated brains in an injected dose-dependent manner. Scale bar, 5 mm.

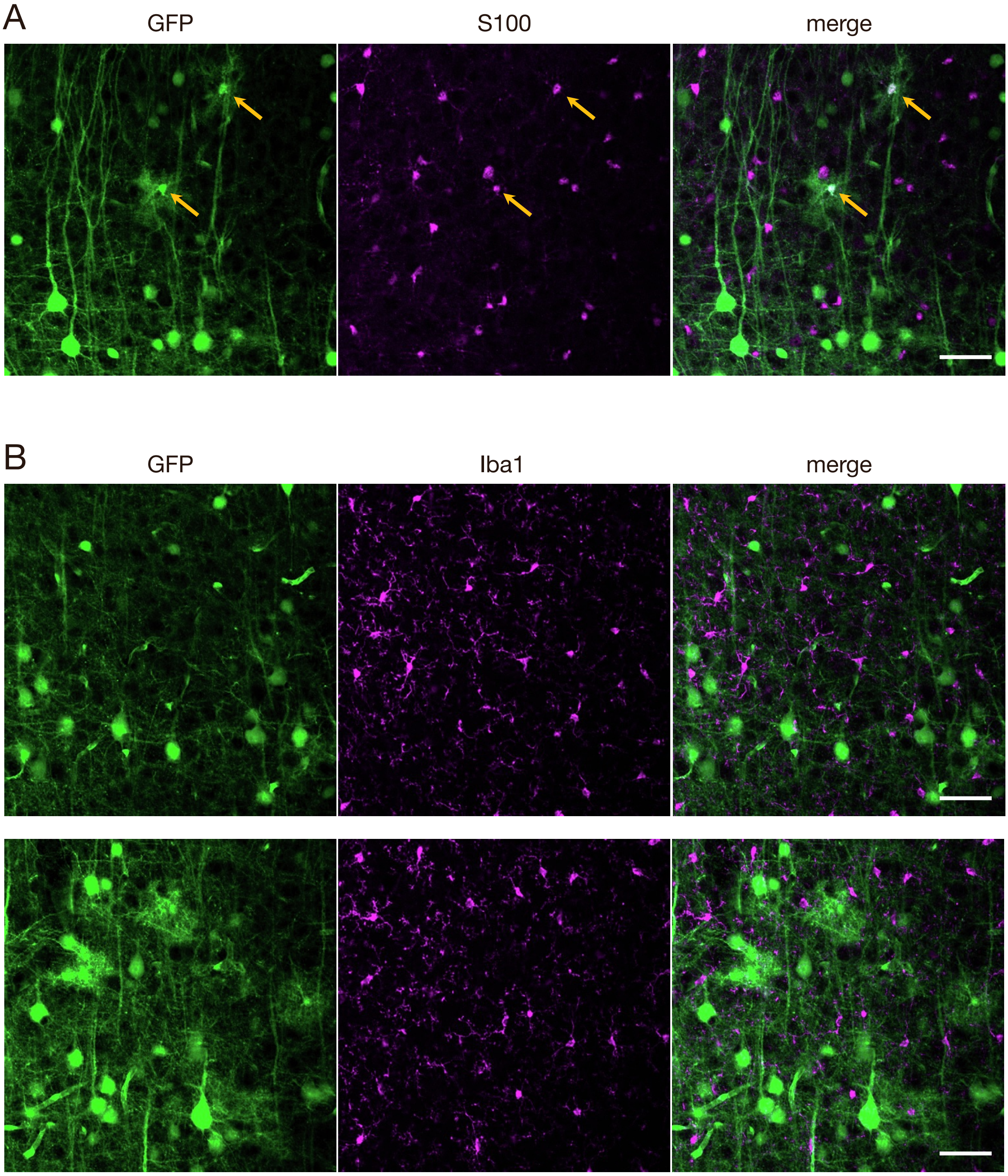

Figure S2. Intravenous injection of BR1N transduces astrocytes, but not microglia. C57BL/6 mice received intravenous injection of BR1N expressing GFP under the control of the CBh promoter at a dose of 1×10^{11} vg/mouse. Three weeks after the AAV injection, sagittal sections of the brain were immunolabeled for astrocyte marker S100 (**A**) or microglia marker Iba1 (**B**). Yellow arrows show GFP and S100 double positive cells. Scale bar, 100 μm.

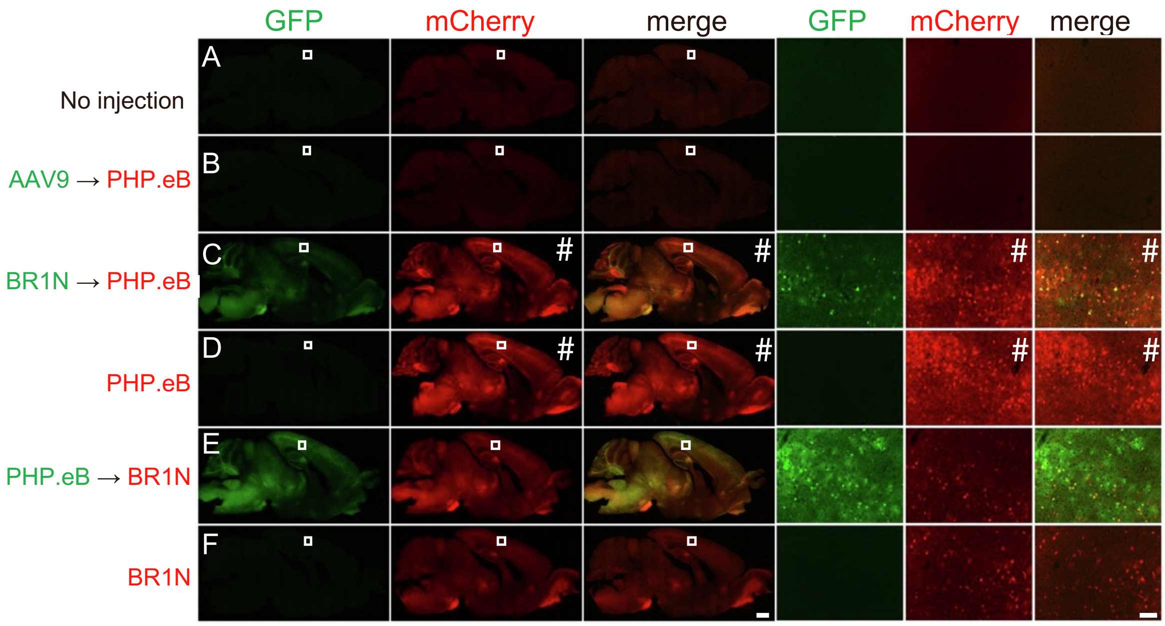

Figure S3. No cross-reaction of NAbs between AAV9 and BR1N with 1-week interval. C57BL/6 mice received systemic AAV injections twice with a 1-week interval: first injection (AAVs expressing GFP) was aimed to produce NAbs, whereas second injection used different capsid AAVs expressing mCherry to test the cross-reactivity of the NAbs produced by the 1st AAV injection. Mice were treated as described on the left side of the panels. GFP and mCherry fluorescence and overlayed images of sagittal brain sections are presented (three columns of the left side), from which square regions in the cerebral cortex are magnified (three columns of the right side). Second systemic injection of PHP.eB caused much greater mCherry expression than BR1N injection. To avoid halation of the fluorescence, mCherry fluorescence in panels marked with hash (#) was obtained using a shorter exposure time. Scale bars: 1 mm (left) and 100 µm (right).

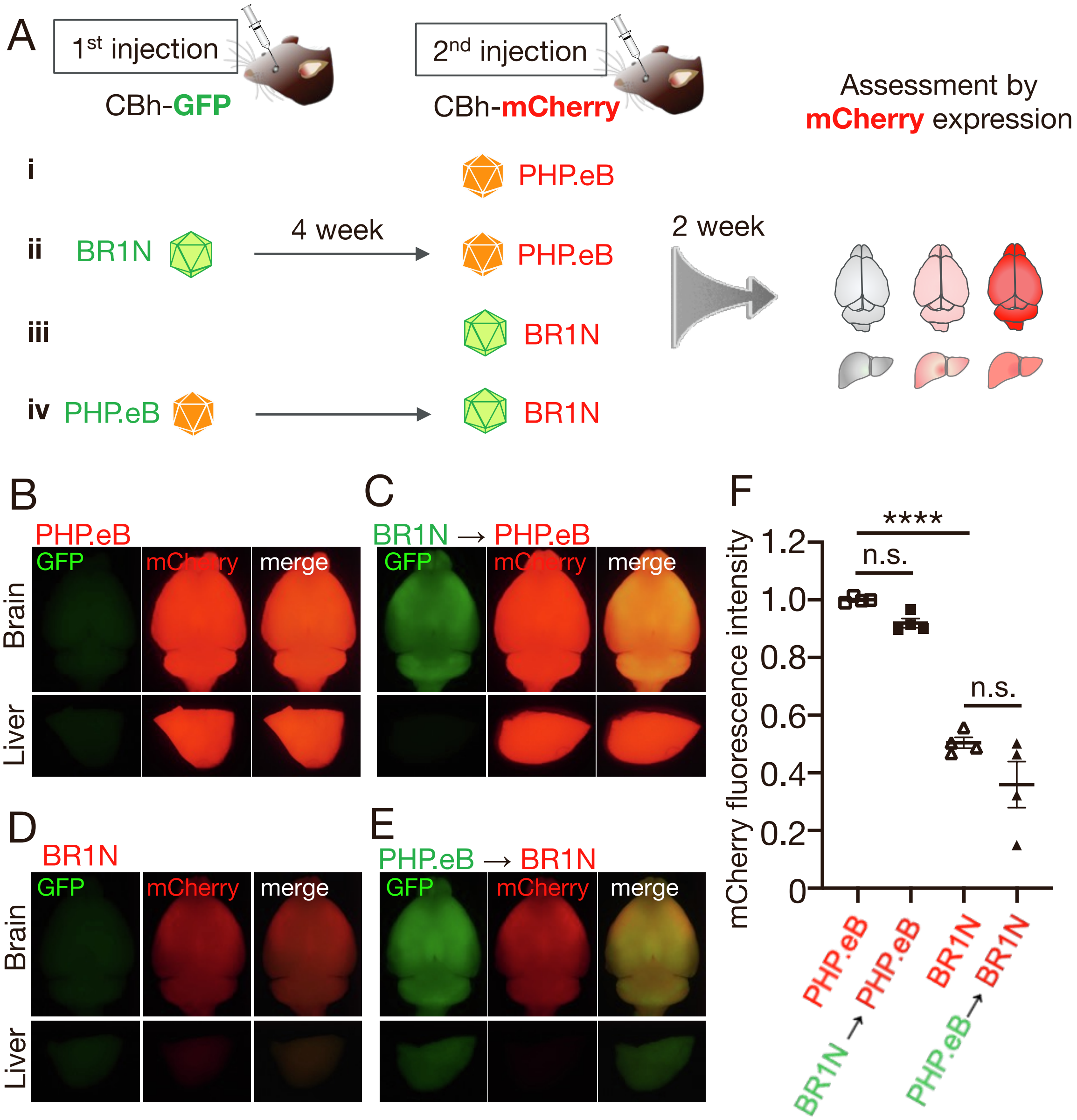

Figure S4. Absence of cross-reaction of NAbs between AAV9 and BR1N with 4-week interval. (**A**) Diagram showing the experimental procedure. C57BL/6 mice received systemic AAV injections twice with a 4-week interval: first injection (AAVs expressing GFP) was aimed to produce NAbs, whereas second injection used different capsid AAVs expressing mCherry to test the cross-reactivity of the NAbs produced by the 1st AAV injection. The cross-reactivity was assessed two weeks after the second injection by mCherry fluorescence intensity. (**B-E**) Representative GFP and mCherry fluorescence images of the whole brain and liver from mice virally treated as described above respective panels. Fluorescence images of whole brains and livers from mice treated with mCherry-expressing PHP.eB

alone (**B**) or mCherry-expressing BR1N alone (**D**) were presented as controls. (**F**) Summarized graph showing the mCherry fluorescence intensity from whole brains. Asterisks show statistically significant differences ($n = 4$ mice per group, ****$P<0.0001$ by one-way ANOVA with Bonferroni's post hoc test. n.s., not significant). All error bars show SEM.

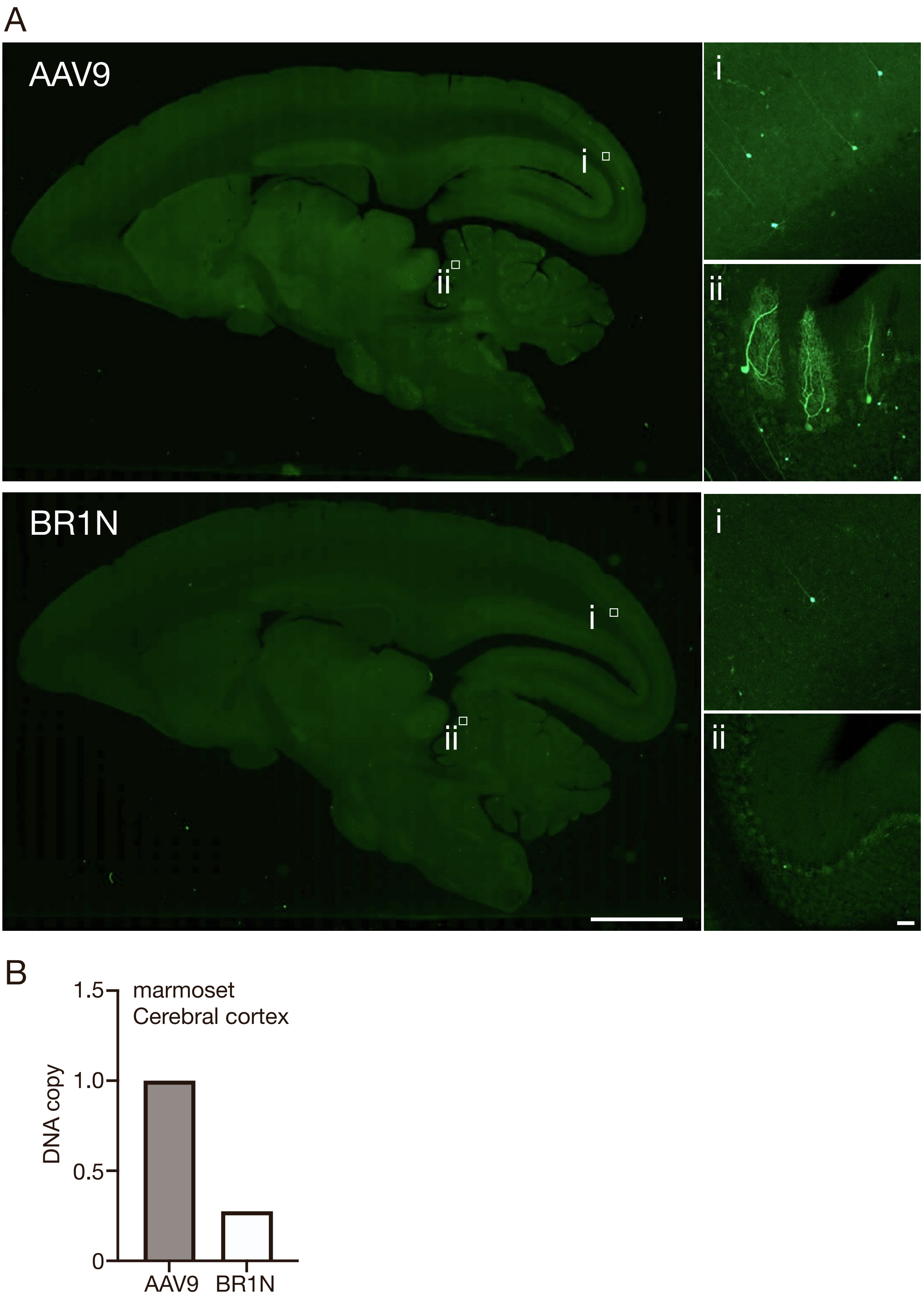
A
AAV9
i
ii
BR1N
i
ii
B
1.5
1.0
0.5
0
marmoset
Cerebral cortex
DNA copy
AAV9
BR1N

Figure S5. No enhanced BBB penetration of intravenously infused BR1N in marmoset. Male marmosets at 1.4 years of age received intravenous injection of AAV9 or BR1N (5×10^{11} vg/kg) through the femoral vein. Four weeks after the injection, treated animals were sacrificed for histological analysis and quantitative real-time PCR. (**A**) Low-power images of sagittal brain sections from marmosets treated with AAV9 or BR1N (left). Images on the right (i and ii) are magnification of square regions in left low-power images. Scale bars: 5 mm (left) and 100 µm (right). (**B**) Markedly less AAV genome DNA content in the cortical tissue from BR1N-treated marmoset than in the tissue from the AAV9-treated marmoset. Cerebral cortical tissues from marmosets treated with AAV9 or BR1N were subjected to quantitative real-time PCR.
